# Comparative evaluation of methanolic extracts from black pepper varieties: yield, GC-MS characterization, and antioxidant and antibacterial activities

**DOI:** 10.3389/fbioe.2026.1845332

**Published:** 2026-09-25

**Authors:** Shaimaa A. M. Abdelmohsen, Arooj Anees, Mohsin Javed, Sana Mansoor, Misbah Umar, Urooj Fatima, Zafar Iqbal, Muhammad Faizan, Chi-Hsien Liu, Syed Kashif Ali, Shahid Iqbal, Sajid Mahmood, Meznah M. Alanazi

**Affiliations:** 1 Department of Physics, College of Science, Princess Nourah bint Abdulrahman University, Riyadh, Saudi Arabia; 2 Department of Chemistry, School of Science, University of Management and Technology, Lahore, Pakistan; 3 Department of Chemistry, University of Engineering and Technology, Lahore, Pakistan; 4 Senior Scientific Officer, Applied Chemistry Research Centre, PCSIR Laboratories Complex, Lahore, Pakistan; 5 Department of Chemical and Materials Engineering, Chang Gung University, Taoyuan, Taiwan; 6 Department of Physical Sciences, Chemistry Division, College of Science, Jazan University, Jazan, Saudi Arabia; 7 Engineering and Technology Research Center, Jazan University, Jazan, Saudi Arabia; 8 Department of Chemical and Environmental Engineering, University of Nottingham, Ningbo, China; 9 Department of Mathematics and Natural Sciences, Gulf University for Science and Technology, Kuwait City, Kuwait

**Keywords:** antibacterial, antioxidant, piperine, Soxhlet extraction, yield

## Abstract

One of the most commonly used culinary spices, black pepper (*Piper nigrum L.*) is also a rich source of bioactive phytochemicals. The present study aims to investigate the oleoresin extracted from black and white pepper using a Soxhlet extraction system and describe its physicochemical characterization, GC-MS analysis, and antioxidant and antibacterial activity evaluation. The antioxidant capacity was determined by the 2,2-diphenyl-1-picrylhydrazyl (DPPH) free radical scavenging assay, and the antibacterial activity was determined by the agar well diffusion method. The extract had the highest antibacterial activity against Gram-positive *Bacillus subtilis*, with zones of 20 mm and 16 mm for black and white pepper extracts, respectively. The antioxidant activity of both extracts was found to be high, with DPPH radical scavenging activities of 86.80% for black pepper extract and 87.62% for white pepper extract. The oleoresin yield was 4.2% for black pepper and 3.2% for white pepper. GC–MS analysis showed a variety of phytochemical constituents such as alkaloids, terpenoids, fatty acids, and sterols. Major alkaloids of black pepper were found as piperine (8.54%) and yohimbine (12.27%), while the sterols were the most abundant class of compounds present in white pepper (28.67%). The total alkaloids present in the oleoresins of black pepper and white pepper were approximately 35%. This indicated that the oleoresins may serve as a natural source of biologically active compounds.

## 1 Introduction

The blooming vine that is popularly referred to as black pepper is a member of the Piperaceae family (Nair and Gupta, 2003; Abbasi et al., 2010). Of the 1,000 species in the Piperaceae family, *P. nigrum* L. is one of the most significant cultivated species because of its economic significance (Bhat et al., 1995). Pepper is frequently used in curry masalas and is a key ingredient in Ayurvedic, folk, and traditional medicine treatments (Rani et al., 2013). The presence of piperine, including its many isomers, makes *Piper nigrum* L. extremely valuable (Zaveri et al., 2010). Due to its many therapeutic benefits, *P. nigrum* is frequently used in Indian, Latin American, and West Indies medicine (Scott et al., 2008). Black pepper can be used as a biocontrol agent, a medication, a preservative, as well as in human diets (Srinivasan, 2007; Hussain et al., 2011). In addition to its nutritional benefits, black pepper’s physiological actions make it a useful agent for preventing chronic illnesses (Sruthi et al., 2013). Black pepper is also used for its antibacterial, anti-inflammatory, anti-cancer, and gastroprotective properties (Butt et al., 2013).

The main components that give white and black pepper their spicy flavor are oleoresin, piperine, and essential oil (Tainter and Grenis, 2001). One of the pepper’s main bioactive ingredients, piperine, is a nitrogen-containing molecule that gives pepper its pungency (Ravindran and Kallupurackal, 2012). Piperine can be sensitive to light and oxygen (Parthasarathy et al., 2008). The Piperaceae family contains a wide range of piperine concentrations. For instance, black pepper has been reported to contain approximately 2.0% and 7.4% piperine, respectively, while white pepper has been discovered to contain up to 9.0% piperine (Peter, 2006; Ravindran, 2003). Overall, pepper’s worth is determined by its flavor and pungency, which are typically ascribed to the presence of oleoresin, the piperine alkaloid, and the essential oils (Gorgani et al., 2017).

One of pepper’s primary ingredients, pepper oleoresin, is an oily material that is extracted from pepper powder using organic solvents. Pepper oleoresin is a dark green, viscous liquid that tastes and smells peppery. It tastes harsh and spicy with a touch of clove and lemon. Its primary constituents are piperine and volatile oil, which contain pepper’s strong stimulant compounds and other fragrant components (Nagavekar and Singhal, 2018). Because of its unique aroma, pepper oleoresin is used in a wide range of dishes, such as meats, sauces, chutneys, soups, and snacks. Piper oleoresin’s widespread usage in the food processing sector is made possible by its convenient storage and mobility. The primary pungent component of pepper oleoresin, piperine, has been shown in several studies to offer functional health advantages. According to the reports, piperine has anti-inflammatory, anti-cancer, antibacterial, and anti-diabetic effects (Bui et al., 2020; Tedasen et al., 2020; Zarai et al., 2013; Zinjarde et al., 2011). Additionally, piperine has been shown to have a strong inhibitory effect on the severe acute respiratory syndrome coronavirus 2 (COVID-19) because of its high affinity for the RNA-binding site (Choudhary et al., 2020). The majority of expensive, highly processed goods, such as pepper essential oil, pepper oleoresin, and pepper capsules, are imported into Pakistan, whereas China mostly processes and manufactures primary pepper products (Olalere et al., 2017; Haq et al., 2021). The chosen technique and solvent can be used to extract these advantageous chemicals.

Subcritical water extraction (Hassas-Roudsari et al., 2009), continuous subcritical water extraction (Gamiz-Gracia and De Castro, 2000; Jimenez-Carmona et al., 1999; Ayala and De Castro, 2001), traditional solvent extraction (Zarai et al., 2013; Shaikh et al., 2006; Shingate et al., 2013), supercritical fluid extraction (Hamrapurkar et al., 2011; Lenucci et al., 2015), and hydrotropic extraction are some of the several extraction methods that have been previously documented. According to other research, of five distinct extraction techniques, the traditional Soxhlet method produces the best overall results (Prytula and Paviostathis, 1996). Thus, in this investigation, Soxhlet extraction using dry and ground sediment was selected. It is well known that different types of extractants have varying reactions and capacities to draw out the bioactive substances found in food samples. Pepper can be extracted using organic solvents such as ethanol, acetone, or chlorinated hydrocarbons (Marion, 1960), dichloromethane (Raman and Gaikar, 2002), petroleum ether and chloroform (Kolhe et al., 2011), a combination of solvents such as n-hexane, ethyl acetate, and glacial acetic acid, as well as ethanol and water (Santamaria et al., 2000), according to earlier publications. Because the various solvents have distinct effects and produce different outcomes, the findings from these experiments varied. This study aims to ascertain the physicochemical, antibacterial, and antioxidant properties of pepper oleoresin extracted from black pepper.

Although numerous studies have investigated black pepper constituents, direct comparative evaluation of methanolic oleoresin extracts from black and white pepper varieties with respect to extraction yield, GC-MS chemical profiling, antioxidant activity, antibacterial activity, and physicochemical characteristics remains limited. The aim of the present study was to compare these parameters on a comprehensive basis using a single extraction method, offering valuable information about the potential application of pepper oleoresins in food and pharmaceutical fields.

## 2 Experimental work

### 2.1 Soxhlet extract preparation

Approximately 500 g of the dried black pepper was taken and crushed into a ground form. Then, approximately 1000 mL of methanol was added as a solvent. Following this, the extract was separated using a funnel and a Soxhlet extraction apparatus. Filtration was done using Whatman filter paper No. 41. Following distillation, an extract was made (Equation 1) and continued for additional examination, which we assessed quantitatively for antibacterial and antioxidant properties and qualitatively using several assays.

#### 2.1.1 Determination of extraction yield

$$Yield\;\left(\%\right)=\frac{Weight\;of\;extract}{Weight\;of\;sample}\times 100,$$ (1)

where

- Weight of the extract = W_2_ − W_1_
- W_1_ = weight of empty beaker
- W_2_ = weight of beaker containing extract

The calculated yields were subsequently reported in Section 3, Results and discussion.

### 2.2 Antioxidant activity

Using a UV-visible spectrophotometer to monitor 2,2-diphenyl-1-picrylhydrazyl (DPPH) solution with methanol is a very accurate, straightforward, and efficient method. Because of absorption by an oxidant, the hue changed from purple to yellow. The capacity of the stable free radical DPPH to donate hydrogen was used to measure antioxidant activity. The antioxidant activity of methanolic extract of *Piper nigrum* was determined using the DPPH radical UV-Visible spectrophotometer (Brand-Williams et al., 1995). A UV-Visible spectrophotometer (Model 1700, Shimadzu, Japan) was the device used. Methanol was used as the solvent, and DPPH was used as a solute to create the DPPH solution, which was prepared by dissolving 4 mg of DPPH in 100 mL of methanol. The solution concentration was approximately 0.10 mM. The mixture was wrapped in aluminum foil. Various concentrations of 10 µm, 30 µm, and 50 µm were prepared using the sample. Next, in separate test tubes, 3 mL of the DPPH:methanol solution was combined with the various sample concentrations. Because there were no antioxidants present, the blank had the maximum absorbance of 0.982. Following this, a UV–visible spectrophotometer was used at room temperature in a light-protected location to measure the absorbance of the blank and the succeeding solutions at 517 nm. The accompanying condition was used to calculate the % inhibition.

$$\text{Percentage inhibition}=\left(\text{Absorbance of control}-\text{Absorbance of sample}/\text{Absorbance of control}\right)\times 100$$

### 2.3 Antibacterial activity of varieties of *Piper nigrum*

Various bacterial strains, including Gram-negative bacteria such as *Escherichia coli* and Gram-positive bacteria such as *Staphylococcus aureus* and *Bacillus subtilis,* were tested for antibacterial activity. In Petri dishes containing a sample of *P. nigrum* methanolic extract, imipenem (0.1%) was employed as the standard against bacterial strains. These strains of bacteria were gathered at the Lahore laboratory. The disc diffusion method was used to determine antibacterial activity. Petri plates were coated with a nutritious agar mixture and allowed to cool. Wells of 6 mm diameter were made in nutrient agar plates and filled with the respective extracts. Then, the bacterial suspensions of Gram-negative bacteria such as *E. coli* and Gram-positive bacteria such as *S. aureus* and *B. subtilis* were introduced to the Petri plates. After that, these were incubated for a 24 h period at 37 °C to test for bacterial strains (Clinical and Laboratory Standards Institute (CLSI) guidelines). When comparing different types of *P. nigrum* extract, the zone of inhibition was calculated by measuring the disc’s diameter in millimeters from the disc plate.

### 2.4 GC–MS analysis of black and white pepper methanolic extract

The investigation of oleoresin was performed on a gas chromatograph (GC-14A) combined with a mass spectrometer for information preparation. A PEG narrow-bore column (25 m × 0.2 mm) was utilized for oleoresins. The injector and detector temperatures were maintained at 150 °C and 250 °C, respectively. The flow rate of the carrier gas (helium) was 1.5 mL/min, and the split proportion of the segments was based on their retention time compared to those obtained from oleoresins analyzed under similar conditions. Electron ionization was used at 70 eV, and the scan range was 30–550 m/z. The temperature of the MS transfer line was adjusted to 200 °C, and the injector temperature was maintained at 180°. The retention time of unknown compounds was identified by comparison, and further interpretations were based on the National Institute of Standards and Technology (NIST) library system.

### 2.5 Proximate analysis

The moisture, crude protein, ash, crude fiber, crude fat, and carbohydrate contents of black and white pepper samples were assessed using the conventional Association of Official Analytical Chemists (AOAC) methodologies. All assays were conducted in triplicate, and findings are presented as % composition based on the dry weight.

### 2.6 Statistical analysis

Every experiment was carried out three times. The mean ± standard deviation is used to represent the results. Data were analyzed using Microsoft Excel. DPPH scavenging equation and measurement of the zone of inhibition (mm) were used to calculate the antioxidant activity and antibacterial activity, respectively.

## 3 Results and discussion

### 3.1 Chemical analysis of methanolic extracts of black pepper

The methanolic extract of *Piper nigrum* was subjected to qualitative phytochemical screening, and the alkaloids, glycosides, terpenoids, saponins, and tannins were found (Table 1). The occurrence of these secondary metabolites indicates that methanol is a good solvent for bioactive compounds, which are responsible for the medicinal properties of black pepper. The biological activities observed in the present study can be attributed to these phytochemicals, which have been reported to possess antimicrobial, antioxidant, and anti-inflammatory properties. The results are in accordance with previous reports that identified alkaloids, tannins, saponins, terpenoids, and glycosides in methanolic extract of *P. nigrum* and attributed the therapeutic potential to these bioactive constituents (Nahak and Sahu, 2011; Damanhouri, 2014). Thus, the phytochemical profile of this study confirmed the traditional use of black pepper as a medicinal plant and its potential as a source of natural therapeutic agents.

**TABLE 1:** Qualitative phytochemical results of the *P. nigrum* methanolic extract.

Phytochemical constituent	Identification test	Methanol
Saponins	Foam test	+
Terpenoids	Salkowski	+
Glycosides	Borntrager’s test	+
Tannins	Ferric chlorides	+
Alkaloids	Dragendorff’s test	+

(+) shows the occurrence of constituent (−) show the constituent is absent.

### 3.2 Quantitative analysis of *Piper nigrum* oil

#### 3.2.1 *Piper nigrum* nutritional analysis

Table 2 shows the proximate composition of black and white pepper. Protein, dietary fiber, moisture, ash, and fat were present in appreciable amounts in both of the samples. The moisture content (9.93%) and ash content (13.10%) were higher in black pepper than in white pepper, while the protein content (10.11%) was higher in white pepper. Both samples had similar amounts of fat, fiber, and carbohydrates. The findings suggest that *P. nigrum* is a nutritionally valuable spice that can be used to provide other nutritional benefits in addition to flavor.

**TABLE 2:** Nutritional analysis of black pepper.

Sr. No	Constituents	Black pepper Amounts	White pepper Amounts
1	Moisture	9.93%	7.87%
2	Ash	13.1%	7.02%
3	Protein	8.23%	10.11%
4	Fat	4.40%	4.40%
5	Fiber	6.85%	6.85%
6	Carbohydrate	12.62%	12.62%

The nutritional composition observed is in general agreement with the previous studies that reported black pepper is an excellent source of proteins, dietary fiber, and minerals, along with bioactive compounds. The minor differences in the proximate composition can be explained by cultivar, geographical origin, harvesting stage, processing techniques, as well as the environmental factors (Parthasarathy et al., 2008; Ravindran and Kallupurackal, 2012).

### 3.3 GC–MS analysis of black pepper oil

Gas chromatography–mass spectrometry (GC–MS) was used to analyze the chemical composition of black pepper oleoresin. The chromatogram (Figure 1) showed the presence of several phytochemical compounds that were identified by comparing the retention time and mass spectra with reference compounds present in the NIST mass spectrum library. The isolated compounds are the major volatile and semi-volatile components of the black pepper oil (Table 3). GC–MS analysis showed that black pepper oleoresin contains a variety of bioactive phytochemicals, mainly alkaloids, terpenoids, fatty acids, and alcohol derivatives.

**FIGURE 1:**
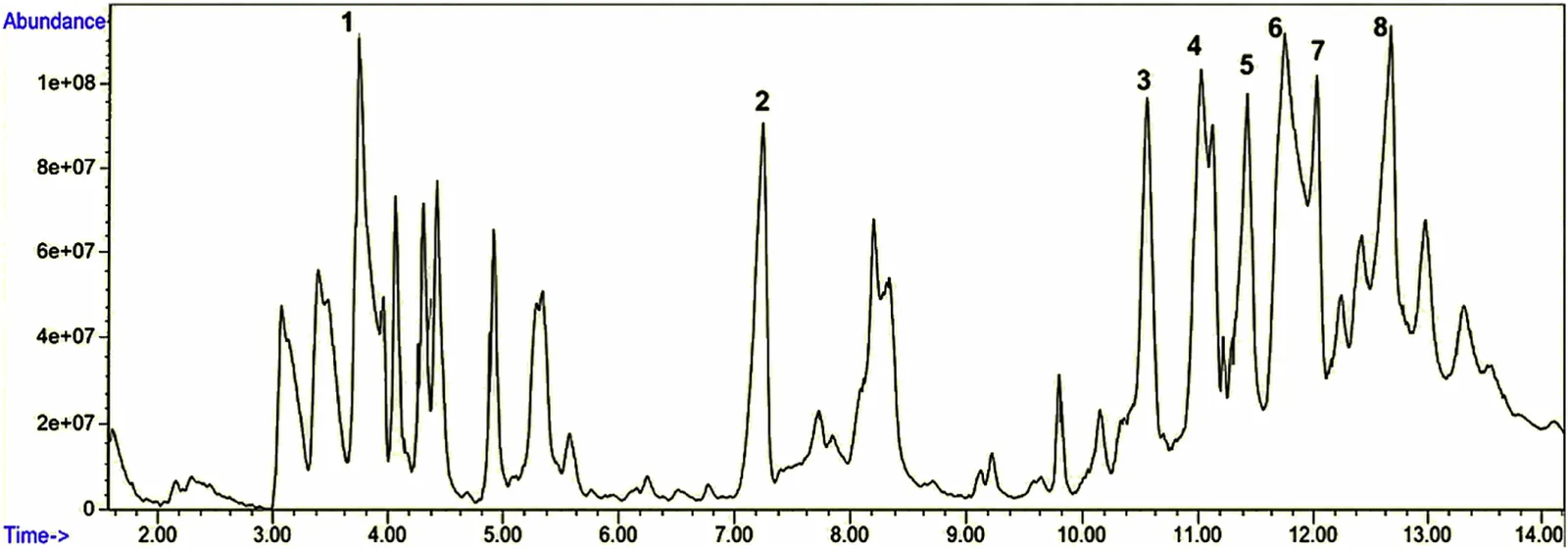
GC–MS of the black pepper oil.

**TABLE 3:** Compounds present in the black pepper oil.

Peaks	Retention time	Compound name	Class of compound	Molecular formula	Molecular weight g/mol	Peak area %
Peak 1	3.759	Caryophyllene	Sesquiterpene	C_15_H_24_	204.35	4.26
Peak 2	7.245	2,4-Decadienamide, N-isobutyl-	n-acyl amines	C_14_H_25_NO	223.35	3.71
Peak 3	10.562	Thymol	Monoterpene	C_10_H_14_O	150.2	4.63
Peak 4	11.015	Ethchlorvynol	Carbinol	C_7_H_9_ClO	144.5	7.21
Peak 5	11.420	Oleic acid	Fatty acid	C_18_H_34_O_2_	282.2	4.61
Peak 6	11.722	Piperine	Alkaloids	C_17_H_19_NO_3_	285.3	8.54
Peak 7	12.000	Tioconazole	Benzyl ethers	C_16_H_13_Cl_3_N_2_OS	387.7	3.23
Peak 8	12.628	(E)-9-(Benzo[d] dioxol-5-yl)-(pyrrolidin-1-yl)non-8-en-1-one (Nair and Gupta, 2003; Bhat et al., 1995)	Alkaloids	C_20_H_27_NO_3_	329.4	7.69

GC–MS analysis revealed that black pepper oleoresin contains a diverse range of bioactive phytochemicals, predominantly alkaloids, terpenoids, fatty acids, and alcohol derivatives. Piperine (8.54%), *(E)-9-(Benzo[d] dioxol-5-yl)-(pyrrolidin-1-yl)non-8-en-1-one* (7.69%), and ethchlorvynol (7.21%) were identified as major constituents (Nair and Gupta, 2003; Bhat et al., 1995). Monoterpenes (4.63%), oleic acid (4.61%), and sesquiterpenes (4.26%) were found in relatively high concentrations, while caryophyllene oxide and the other fatty acids were present in relatively lower concentrations. Additionally, n-acyl amines and benzyl ether derivatives were also detected. The high percentage of alkaloids, especially piperine, is consistent with the results of the previous studies, which have identified piperine as the main bioactive compound with many of the pharmacological properties of black pepper, such as antioxidant and antimicrobial activities and contributing to the characteristic pungent flavor of black pepper (Parthasarathy et al., 2008; Ravindran, 2012).

### 3.4 GC–MS analysis of white pepper oil

The chemical composition of white pepper oleoresin was characterized by gas chromatography–mass spectrometry (GC–MS). The chromatogram (Figure 2) showed several phytochemical components that were identified based on their retention time and mass spectra matching with the NIST mass spectrum library of reference compounds.

**FIGURE 2:**
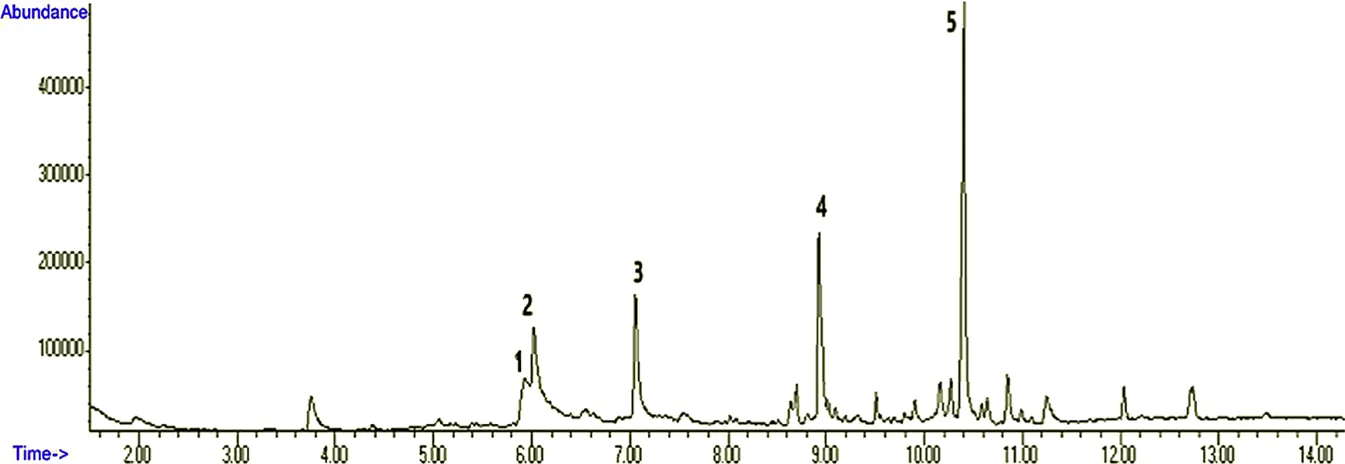
GC–MS of the white pepper oil.

The GC–MS analysis revealed that white pepper oleoresin is rich in various bioactive compounds, mainly alkaloids, sterols, and hydrocarbons. The alkaloids and the sterols were the major chemical classes of the extract, represented by the major constituents identified: yohimbine (12.27%) and cholesta-3,5-diene (28.67%) (Table 4). A relatively high level of octadecanenitrile was also found (10.28%), and caffeine, a purine alkaloid, was found at 2.91%. Very small quantities (0.12%) of dodecane (1,1′-oxybis-) were found. Sterols and alkaloids are predominant; they indicate that white pepper has high biological potential (Parthasarathy et al., 2008; Gorgani et al., 2017).

**TABLE 4:** Compounds present in the white pepper oil.

Peaks	Retention time	Compound name	Class of compound	Molecular formula	Molecular weight g/mol	Peak area %
Peak 1	5.825	Dodecane, 1,1′-oxybis-	Ether	C_24_H_50_O	354.6	0.12
Peak 2	5.928	Caffeine	Purines	C_8_H_10_ N_4_O_2_	194.19	2.91
Peak 3	7.058	Octadecane nitrile	Acyclic alkanes	C_18_H_35_N	265.4	10.28
Peak 4	8.931	Yohimbine	Indole alkaloid	C_21_H_26_N_2_O_3_	354.44	12.27
Peak 5	10.405	Cholesta-3,5-diene	Sterol	C_27_H_44_	368.6	28.67

### 3.5 Determination of the antioxidant activity of *Piper nigrum*

*Piper nigrum* has been used medically to treat many diseases for thousands of years. It reduces the formation of oxidative species by acting as a natural antioxidant. Antioxidant activity was determined using DPPH, a stable free radical. Both synthetic and natural antioxidants convert DPPH free radicals into the yellow diphenylpicrylhydrazine. In this work, antioxidant activity was determined for the extract obtained from *P. nigrum* varieties by using DPPH (Table 5). Methanolic black and white pepper extracts indicated the strongest cancer-preventive agent action of 86.80% and 87.62% at 50 μg/mL. As the concentration increased to 50 μg/mL, the percentage inhibition also increased. The strong antioxidant activity observed in both extracts may be attributed to piperine, phenolic compounds, and terpenoid constituents. Similar antioxidant activities of *P. nigrum* extracts have been reported by Zarai et al., 2013 and Haq et al. (2021), who demonstrated significant radical scavenging activity associated with that of the piperine-rich extracts.

**TABLE 5:** Antioxidant activity of methanolic extract of black pepper and white pepper.

Sr. No	Concentration/mL	Black pepper		White pepper	
		Absorbance	% inhibition	Absorbance	% inhibition
1	10	0.5260	47.40%	0.4933	50.67%
2	30	0.2044	79.56%	0.1987	80.13%
3	50	0.1320	86.80%	0.1238	87.62%

### 3.6 Antibacterial activity of varieties of *Piper nigrum*

Dried varieties of *Piper nigrum* extract were tested by the disc diffusion method against three strains of bacteria: Gram-positive (+ve) *Bacillus subtilis* and *Staphylococcus aureus* and Gram-negative (−ve) *Escherichia coli*. Methanolic extract of *P. nigrum* indicated high antibacterial activity. Standard imipenem showed antibacterial activity in the presence of methanolic extract of varieties of *P. nigrum.* The temperature required for antibacterial activity was 37 °C. The higher antibacterial activity observed against *Bacillus subtilis* may be associated with the disruption of bacterial membrane integrity by alkaloids and terpenoid compounds present in extracts. Similar observations were reported by Rani et al. (2013). The zone of inhibition for methanolic extract of varieties of *P. nigrum* against bacterial species is given in Figure 3.

**FIGURE 3:**
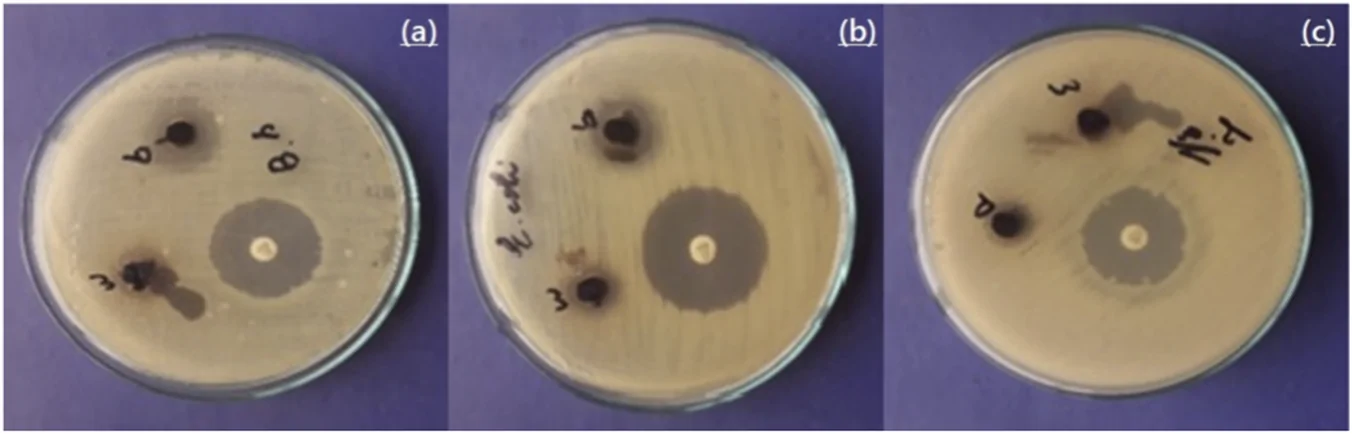
Result of antibacterial activity for methanolic extract of varieties of *Piper nigrum* against **(a)** *Escherichia coli,* **(b)** *Bacillus subtilis,* and **(c)** *Staphylococcus aureus*.

*P. nigrum* showed the maximum zones of inhibition against *Bacillus subtilis* and *Escherichia coli*: 20 mm for black pepper and 16 mm for white pepper. Meanwhile, *Staphylococcus aureus* showed the minimum zone of inhibition. Both black and white pepper extracts contain phenolic contents, but it’s comparatively higher in black pepper extract. The sugars readily dissolve in the moisture of the agar gel, and the phenolic hydroxyl (-OH) groups in the pepper oil bond with the methanol solvent, preventing the active antibacterial compounds from properly diffusing into this aqueous sugar matrix.

### 3.7 Percentage yield of black pepper

The percentage yield of black pepper is calculated by using the following formula (Equation 2).

Weight of dried beaker = W_1_ = 52.54 g

Weight of beaker with oil = W_2_ = 73.55 g

Weight of oil = W_2_ - W_1_ = 21.01

$$\text{Percent yield}=\frac{21.01}{500}X100=4.2\%.$$ (2)

The yield of black pepper oil obtained was 4.2%.

### 3.8 Percentage yield of white pepper

The percentage yield of white pepper is calculated by using the following formula (Equation 3).

Weight of dried beaker = W_1_ = 52.54 g

Weight of beaker with oil = W_2_ = 68.83 g

Weight of oil = W_2_ - W_1_ = 16.29

$$\text{Percent yield}=\frac{16.29}{500}X100=3.2\%.$$ (3)

The yield of white pepper oil obtained was 3.2%.

## 4 Conclusion

This study demonstrated that methanolic oleoresins of both the black and white pepper are rich sources of biologically active phytochemicals with promising antioxidant and antibacterial properties. The oleoresin content of black pepper was higher than that of white pepper. The GC–MS analysis revealed the presence of a variety of bioactive compounds such as alkaloids, terpenoids, sterols, and fatty acids. The major alkaloids in black pepper were found to be piperine, and the major constituents of white pepper were sterols. The extracts had high DPPH radical scavenging activity (>86%) and significant antibacterial activity, especially against *Bacillus subtilis*, suggesting that the extracts can be used as natural antioxidant and antimicrobial agents. The results confirm the traditional uses of *Piper nigrum* for its medicinal properties and suggest its use in the pharmaceutical, food, and nutraceutical sectors. Further investigations should be conducted involving isolation of individual bioactive compounds and *in vivo* investigations to better understand their therapeutic efficacy and mechanisms of action.

## Data Availability

The original contributions presented in the study are included in the article/supplementary material; further inquiries can be directed to the corresponding author.
